# The Impact of Diabetes Type 2 in the Pathogenesis of Benign Prostatic Hyperplasia: A Review

**DOI:** 10.1155/2009/818965

**Published:** 2009-11-09

**Authors:** K. Stamatiou, M. Lardas, E. Kostakos, V. Koutsonasios, E. Michail

**Affiliations:** Urology Department, Tzaneion General Hospital, 2 Salepoula street, 18536 Piraeus, Greece

## Abstract

*Introduction*. Clinical observation of larger prostate glands in men with diabetes mellitus type 2 led some investigators to hypothesize that an association between these two conditions exists. In fact, both diseases are very common in men as they age and seem to be sharing similar epidemiologic features. Several studies examining the above hypothesis were yielded. *Aim*. The purpose of this paper is to summarize the existing literature focusing on the coexistence of BPH and diabetes mellitus type 2 and to elucidate whether or not an association among these conditions exists. *Methods*. We identified studies published from 1990 onwards by searching the MEDLINE database of the National Library of Medicine. Initial search terms were *benign prostatic hyperplasia, epidemiology*, and *risk factor,* combined with *diet hyperinsulinemia, and diabetes mellitus type 2. Results*. Diabetes mellitus type 2 and hyperinsulinemia are quite common conditions and often coexist with BPH. There are several studies (observational, epidemiological, and experimental) examining the association between them in literature. *Conclusion*. Evidence suggests that an association between BPH and diabetes through a common pathogenic mechanism is possible. The specific pathway interfering in the development of both conditions is still poorly investigated; thus, the exact relationship of BPH to diabetes remains unclear.

## 1. Introduction

Benign prostatic hyperplasia (BPH) is the most common benign tumor in men, and its incidence is age related. It is a significant public health problem, globally, affecting 50% of men aged 60 years or older. BPH represents a pattern of unregulated but nonmalignant growth characterized by an increase in prostate epithelial and stromal cells, especially the latter. The etiology of BPH is still largely unresolved, but multiple partially overlapping and complementary systems (nerve, endocrine, immune, and vascular) as well as local factors are likely to be involved [1, 2]. Although the specific pathway remains poorly investigated, it seems that the pathogenetic mechanism is endocrine controlled [2].

Controversy surrounding BPH pathogenesis along with the fact that both BPH and diabetes mellitus type 2 (DM-2) are both high prevalent diseases is posing doubts on the association between these two common diseases. On the other hand, even though BPH and DM-2 are apparently disparate clinical entities, both diseases seem to be sharing similar epidemiologic features, which are possibly connected to a common pathogenic pathway related to aging and diet [3, 4].

## 2. Methods

We identified studies published from 1990 onwards by searching the MEDLINE database of the National Library of Medicine. Initial search terms were *benign prostatic hyperplasia, epidemiology, and risk factor*, combined with *diet, hyperinsulinemia, and diabetes mellitus type 2*. References in the selected publications were checked for relevant publications not included in the Medline/Pubmed search.

## 3. Results

Bourke and Griffin were the first to suggest an association between diabetes mellitus and BPH etiology, based on the higher prevalence of diabetes mellitus among men subjected to prostatectomy than in the general male population [5]. Almost 30 years later, a study by Hammarsten et al. regenerated the scientific interest on the association between these two conditions. They showed that patients with lower urinary tract symptoms (LUTSs) and DM-2 had larger prostate volumes than patients with LUTSs without diabetes mellitus [6]. In addition, Safarinejad and Sarma found a positive association between clinical markers of BPH and diabetes mellitus [7, 8].

Similarly to BPH, DM affects bladder function producing both obstructive and irritative symptoms: the classic triad of obstructive symptoms (difficulty initiating voiding, fullness after voiding, and increased post void residual urine volume) characterizing diabetic cystopathy is also common in BPH. Similarly, frequency and urgency are associated with both DM-induced detrusor instability and BPH. The distinction between LUTS secondary to DM and LUTS secondary to BPH is difficult to disentangle, and often LUTS secondary to DM overlaps LUTS secondary to BPH and vice versa. Moreover, diabetes considerably contributes to the development and the deterioration of LUTS while on the other hand BPH is not always accompanied by symptoms. It is the bothersome of the irritative symptoms that make patients seek medical aid. To our knowledge, these very symptoms are more common among diabetic patients, with 39%–61% of them having some degree of frequency and urgency [9, 10]. As diabetes affects the voiding function, patients with BPH and concomitant DM-2 have more bothersome symptoms and show significantly lower maximum flow rate (Qmax) than nondiabetic BPH patients [11, 12]. For the above reasons, patients with DM-2 are more prone to be diagnosed with BPH and subsequently subjected to prostatectomy than general male population. This fact limits the scientific value of the epidemiologic association between BPH and DM2 through LUTS.

Interestingly, other researchers recognized that from patients with hypertrophy of prostate those with the higher levels of serum glucose (>110 mg/dL) had a considerably higher mean prostate volume in comparison with patients with low levels of serum glucose [13, 14]. Furthermore, Hammarsten and Högstedt comparing anthropologic characteristics with laboratory and clinical data in patients with lower urinary tract symptoms with or without manifestations of the metabolic syndrome demonstrated a further increase in prostate growth rate with the increase levels of serum insulin [15]. This observation was confirmed by Ozden et al. who found considerably higher annual rates of increase in the volume of the transient area diabetics against the patients with low levels of serum glucose [13]. Nandeesha et al. correlated insulin profile parameters with prostate size and found fasting serum insulin and insulin resistance levels significantly higher in nondiabetic BPH cases when compared to controls [16]. Recently, Barnard et al. connected the reduction of growth of stem epithelial prostate cells with the reduction of insulin [17].

Among other possible mechanisms proposed to associate the development of BPH with DM-2 are the increase of the peripheral sympathetic nerve tone and the activity of autonomous nervous system in general due to hyperinsulinemia [18] and hypoxia, due to the decreased blood supply of the prostate deriving from diabetes mellitus-induced vascular damage [19]. Taking in consideration the above information, it could be assumed that abnormalities of glucose homeostasis could play a role in the cause of BPH by influencing the proliferation rate of prostate cells. The specific hypothetic mechanisms are difficult to be identified. Current knowledge supports the idea of a growth-stimulating factor mediating the development and maintenance of the hypertrophic prostate. In fact, insulin is a growth-stimulating hormone that stimulates growth and cell reproduction. The presence of growth factors for prostatic tissue and their role in cellular interactions is known from older studies [18]. In order to evaluate the role of activating factors of BPH growth, Wang et al. investigated the expression of fibroblasts growth factor (fibroblast growth factor2, FGF2) in the prostates of a capable number of rats with experimental-induced diabetes mellitus. They found that the expression of FGF2 was higher in epithelial cells compared to that of the stromal cells of the prostates of the control group; however, the expression of FGF2 was uniformly distributed in the prostates of diabetic group. Interestingly, the peer presence of FGF2 in the stromal and the epithelial layers was consistent with the disproportion in the relation of number of cells of epithelial and stromal layers that is observed in BPH. They also noticed that while the diabetic rats had smaller prostates and lower levels of serum testosterone compared to those of the control group, treatment with insulin increased both the size of prostate and the levels of testosterone [19]. In fact, insulin is a growth hormone that promotes the development and the reproduction of cells. The presence of insulin-like growth factor (IGF) has been known for decades to exist in prostatic tissue. It has been proved also that the prostatic epithelial cells as well as the stromal cells respond to the mitogenic action of IGF through IGF-I receptors. Moreover, it has been proved that the cells of prostatic stromal layer compose and secrete IGF-II as well as conjunctive proteins (IGF-binding proteins/IGFBP) which undergo proteolysis by Prostate Specific Antigen (PSA). Deviations in various elements of IGF system have been observed in the cells of prostatic stroma in BPH as well [20, 21]. Given the dependence of the epithelial as well as the stromal cells from the dihydrotestosterone [22] and the increased activity of IGF-II in the cells of periurethral area [21]—where BPH develops—it could supposed that BPH is promoted by IGF in a male hormone dependent process. Even though such a process has not been completely described, experimental models proved that prostate atrophy induced by androgen deprivation as well as with the effect of antiandrogens and of 5-a-reductase inhibitors is achieved through local growth factors [23, 24]. Particularly for the insulin-like growth factor, experiments proved that its activity is likely to be regulated by androgens. In the absence of them, the number of IGFBP is dramatically diminished while inhibition of testosterone transformation to DHT decreases both IGF-1 receptors as well as the level of IGF-I mRNA [25].

## 4. Conclusion

The exact relationship of BPH to diabetes remains unclear. The specific pathway interfering in the development of both conditions is still poorly investigated. However, evidence suggests that an association between BPH and diabetes through a common pathogenic mechanism through male hormone activity alteration mediated by IGF is possible. The above potentially constitutes the key for the comprehension of the insulin effect and the abnormalities of glucose homeostasis in the development of BPH.
